# *D*‐Allulose as a Low‐Calorie Sweetener: A 30‐Day Randomized, Double‐Blind Study on Gastrointestinal Tolerance and Systemic Safety to Support Its Application in Healthy Diets

**DOI:** 10.1002/fsn3.72042

**Published:** 2026-06-29

**Authors:** Lijuan Qi, Junyu Ning, Jiangli Han, Nan Zhang, Wenjing Zhang, Fangfang Wang, Dan Li, Zinan Li, Yanmin Nie, Haiming Jing, Shan Gao

**Affiliations:** ^1^ Beijing Key Laboratory of Diagnostic and Trace Ability Technologies for Food Poisoning Beijing Center for Disease Prevention and Control Beijing China; ^2^ School of Public Health Capital Medical University Beijing China; ^3^ Department of Cardiology and Institute of Vascular Medicine Peking University Third Hospital Beijing China

**Keywords:** bone mineral density, *D*‐allulose, gastrointestinal tolerance, low‐calorie sweetener, systemic safety

## Abstract

Long‐term safety, tolerance, and population‐specific effects of innovatively fermented *D*‐allulose are lacking in the Chinese population. This study aimed to address these gaps and support its application as a novel food ingredient in China. A 30‐day randomized, double‐blind, parallel‐group design with pre‐post comparison trial was conducted, enrolling 50 healthy Chinese adults (high‐dose group: 36 g/day, 0.6 g/kg body weight, *n* = 26; low‐dose group: 24 g/day, 0.4 g/kg body weight, *n* = 24). Gastrointestinal tolerance was monitored via daily questionnaires; systemic safety was evaluated using hematological tests, serum biochemical tests, urinalysis, fecal analysis, and body composition measurements. The incidence of gastrointestinal symptoms was 48.0%, which were mild, transient, and most frequent on Days 1–3, with no significant intergroup differences. All statistically significant changes in safety indicators remained within normal clinical reference ranges. Compared with baseline, the 30‐day intervention resulted in reduced red blood cell count, hematocrit, and platelet count, as well as elevated mean corpuscular hemoglobin and mean corpuscular hemoglobin concentration in both groups. For serum biochemical parameters, levels of alkaline phosphatase, gamma‐glutamyl transferase, uric acid, total cholesterol, and high‐density lipoprotein cholesterol decreased in both groups, while fasting blood glucose was reduced only in high‐dose group. Notably, high‐dose *D*‐allulose intervention decreased bone mineral density T‐scores in participants aged 35 years and older. All statistically significant alterations in the measured indicators remained within normal clinical ranges and were clinically insignificant. This study provides critical safety data to support the planned approval of *D*‐allulose as a novel food ingredient in China in 2025. Its modulation of hematological and serum biochemical parameters suggests effects on hematopoiesis and glycolipid metabolism. Given the limitations of the 30‐day intervention duration, relatively modest sample size, and restriction to healthy normal‐BMI adults, a provisional safe intake limit of 0.4 g/kg body weight per day is proposed for Chinese adult population, requiring validation in longer‐term and larger‐scale studies.

## Graphical abstract

This graphical abstract presents the key design and findings of a 30‐day trial evaluating the safety of fermented *D*‐allulose in healthy Chinese adults. Fifty eligible adults were randomized to receive either low‐dose (24 g/day, *n* = 24) or high‐dose (36 g/day, *n* = 26) *D*‐allulose for 30 days. Gastrointestinal symptoms (48.0% incidence) were mild, transient, and comparable between groups. All statistically significant changes in hematological, serum biochemical, and bone mineral density parameters remained within normal clinical ranges. Based on these results, a provisional safe intake of 0.4 g/kg body weight/day is proposed for healthy Chinese adults, supporting the regulatory approval of *D*‐allulose as a novel food ingredient in China.

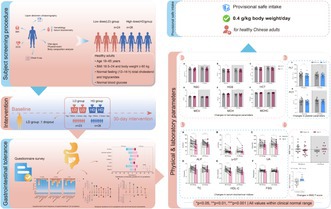

## 1 Introduction

*D*‐allulose, as a novel low‐calorie sweetener, has attracted significant attention in the food industry in recent years. Its unique physical and chemical properties make it an excellent alternative to sucrose. *D*‐allulose may offer multiple benefits for the human body. Regarding blood sugar control, several studies have demonstrated that *D*‐allulose can improve postprandial blood glucose responses. Research shows that *D*‐allulose can reduce post‐meal blood glucose levels, potentially aiding diabetic patients (Hayashi et al. 2010). Iida et al. (2008) found that consuming *D*‐allulose (≥ 5 g) alongside maltodextrin could dose‐dependently lower blood glucose. Noronha et al. (2018) also confirmed in a study with type 2 diabetic patients that adding *D*‐allulose (5 g or 10 g) to a 75 g glucose solution improved the blood glucose response. Concerning weight management and lipid metabolism, *D*‐allulose appears to have promising effects (Sewalt 2016; Itoh et al. 2015). Animal studies indicate that a diet with 5% *D*‐allulose significantly reduces weight gain and fat tissue accumulation in mice. *D*‐allulose (5% diet) can decrease visceral fat and abdominal fat, as well as total body fat in high‐fat diet‐induced obese rats (Chung et al. 2012; Matsuo et al. 2024). In human studies, Tanaka et al. (2020) observed that over a 48‐week trial, daily intake of 15 g *D*‐allulose improved plasma liver enzyme activity, fatty liver scores, and glucose metabolism. Furthermore, studies suggest that *D*‐allulose may also possess liver‐protective properties and regulate gut microbiota (Warda et al. 2025; Adolphus et al. 2025).

With the growing application of *D*‐allulose in food science, concerns over its safety for human consumption have intensified. Current *D*‐allulose safety studies address a wide range of issues, including acute and chronic toxicity, genotoxicity, reproductive and developmental toxicity in animals and the impact on the human body. In animal tests, *D*‐allulose demonstrated low acute toxicity, with an LD_50_ value of approximately 16 g/kg body weight (bw) (Matsuo et al. 2002). In 90‐day chronic toxicity studies, the no‐observed‐adverse‐effect level (NOAEL) was 5000 mg/kg body weight/day (An et al. 2019). Additionally, all genotoxicity tests for *D*‐allulose yielded negative results, indicating that it is neither genotoxic nor carcinogenic (U.S. Food and Drug Administration (FDA) 2017). *D*‐allulose has no negative impacts on reproduction and development in toxicity experiments (Kim et al. 2019; Sa et al. 2022). These research results provide strong evidence for the safety of *D*‐allulose. Our laboratory has also conducted toxicological studies on *D*‐allulose, which was produced using an innovative manufacturing method based on the fermentation of a genetically engineered *Escherichia coli* strain (AS10/CGMCC 27687), including acute oral toxicity in rats and mice, which revealed that *D*‐allulose is virtually non‐toxic. Genotoxicity tests (including the micronucleus test in mouse bone marrow polychromatic erythrocytes, Ames test, and in vitro mammalian cell chromosome aberration test) of *D*‐allulose were all negative. The 90‐day oral toxicity test in rats showed that the NOAEL of *D*‐allulose for female rats was 8000 mg/kg BW and for male rats was 4000 mg/kg BW. The teratogenicity study in rats indicated that the NOAEL of *D*‐allulose was > 5000 mg/kg BW.

Some studies suggest that *D*‐allulose may confer various beneficial effects on the human body; however, its potential adverse effects have also been noted. The most significant adverse effect is gastrointestinal symptoms, particularly diarrhea at elevated doses. A tolerance study found that a single dose of *D*‐allulose ≥ 0.4 g/kg bw can induce diarrhea, typically within 2 h and with a minimum latency of 40 min (Han et al. 2018), likely due to the osmotic effect of unabsorbed *D*‐allulose in the intestines (Food Standards Australia New Zealand (FSANZ) 2023). In another investigation, subjects received an initial dose of 0.4 g/kg bw, followed by increments of 0.1 g/kg bw. Diarrhea was observed in only one subject at a dose of 0.6 g/kg bw; all participants tolerated doses of 0.4 and 0.5 g/kg bw well (Iida 2007). However, existing research on gastrointestinal tolerance to *D*‐allulose in humans has been limited to short‐term or acute exposure studies (Han et al. 2018; Food Standards Australia New Zealand (FSANZ) 2023; Iida 2007), not addressing prolonged or repeated intake effects. While some studies have examined long‐term health impacts over durations of 12 or 48 weeks, the maximum tolerated dose was only 15 g (equivalent to 0.25 g/kg bw) (Hayashi et al. 2010; Tanaka et al. 2020). To the best of our knowledge, no previous study has systematically addressed the key knowledge gaps concerning *D*‐allulose safety in humans, particularly regarding dose range, population specificity, intervention duration, and production method. For dose range, repeated intake of higher doses (≥ 0.4 g/kg bw/day) has not been evaluated beyond acute or single‐dose settings. Regarding population specificity, all existing human tolerance data originate primarily from Japanese and Western cohorts, leaving the Chinese population uncharacterized with respect to potential ethnic differences in gastrointestinal response or systemic metabolism. As for intervention duration, although long‐term studies (12–48 weeks) exist, they employed relatively low daily doses (≤ 15 g, approximately 0.25 g/kg bw) (Hayashi et al. 2010; Tanaka et al. 2020), and no 30‐day repeated‐dose trial has been conducted at 0.4 or 0.6 g/kg bw levels. Finally, concerning production method, the innovative fermentation process using a genetically engineered *Escherichia coli* strain (AS10/CGMCC 27687) yields a *D*‐allulose product with uniquely controlled purity, endotoxin, and protein residue profiles, yet its safety and tolerance profile in humans has never been validated. These gaps collectively limit the evidence‐based application of *D*‐allulose as a novel food ingredient, particularly in the context of China's planned regulatory approval. Therefore, the present study was designed to fill these gaps by conducting a 30‐day randomized, double‐blind, parallel‐group trial in healthy Chinese adults, evaluating two dose levels (0.4 and 0.6 g/kg bw/day) of the fermentatively produced *D*‐allulose.

*D*‐allulose, a low‐calorie fructose epimer with favorable metabolic profiles, is approved as a food ingredient in the United States (GRN No. 693, No. 1029, et al.) (Samyang Corporation 2017; GRAS Associates LLC (Agent for L&P Food Ingredient Co. Ltd) 2021) but remains unauthorized in the European Union due to unresolved long‐term safety data gaps (Turck et al. 2025). This highlights the need for population tolerability evidence to guide global regulatory decisions. In China, rising demand for alternative sweeteners—driven by increased awareness of reduced sugar consumption and high rates of metabolic disorders—highlights the urgent need for population‐specific safety evidence. In July 2025, *D*‐allulose was officially authorized as a novel food ingredient in China through the National Health Commission's Announcement No. 4 (2025). Starting in 2024, this 30‐day intervention trial was designed to address significant evidence gaps regarding the tolerability of *D*‐allulose among Chinese populations prior to its official authorization in China. The findings provided essential scientific support for its approval in China and contributed significantly to the global understanding of this promising sweetener. This work lays important groundwork for addressing regional regulatory disparities (e.g., data deficits within the EU) and ensuring its safe application across global food systems.

## 2 Materials and Methods

### 2.1 Materials Availability

*D*‐allulose in the study was produced through a process utilizing glucose and/or sucrose as primary raw materials. In our previous study (unpublished), we developed an innovative production method based on fermentation by a genetically engineered *Escherichia coli* strain (AS10/CGMCC 27687). This method involved establishing a cell factory by incorporating key enzymes and catalytic elements from the *D*‐allulose synthesis pathway into *E. coli* . The factory's efficiency was then significantly improved through multiple rounds of optimization using metabolic flux analysis, bioinformatics, AI‐assisted high‐performance genetic editing, and online fermentation monitoring. Using this optimized strain (AS10), we developed and refined a robust fermentation process followed by purification and drying, thereby advancing the industrial application of *D*‐allulose.

Quality control assessments were performed on three consecutive industrial batches of *D*‐allulose. Purity ≥ 99% was verified by high‐performance liquid chromatography (HPLC). Endotoxin content was < 10 EU/mg (gel clot method), and residual protein was not detected (Coomassie Brilliant Blue assay). Batch‐to‐batch consistency was confirmed with no significant variability in key quality indicators (Table S1).

The materials used in the study are two intervention beverages: a high‐dose *D*‐allulose beverage and a low‐dose *D*‐allulose beverage, which contained 18 g and 12 *D*‐allulose in 100 mL of drinking water respectively. The *D*‐allulose (CAS No. 551‐68‐8, molecular formula C_6_H_12_O_6_, purity ≥ 99%, white crystalline powder), was supplied by Micro element Synthetic Biotechnology (Beijing) Co. Ltd. (Beijing, China), batch numbers: 20240829 and 20240903, stored at room temperature in a cool, dry place away from light.

### 2.2 Trial Design

A randomized, double‐blind, parallel‐group design with pre‐post comparison clinical trial was designed to minimize the impact of individual baseline variability and precisely capture changes in outcome measures within the same subjects following *D*‐allulose ingestion, thereby evaluating the gastrointestinal tolerance and efficacy of *D*‐allulose in healthy adults. Participants were recruited from Beijing between October 2024 and November 2024, and the trial was conducted from October 2024 to December 2024.

Sample size was determined pragmatically based on similar clinical tolerance studies and regulatory guidelines for novel food ingredients, with gastrointestinal tolerance as the primary feasibility endpoint. No formal power calculation was performed given the exploratory nature of this safety evaluation. A total of 50 participants were enrolled and randomly assigned to the low‐dose group (*n* = 24) or high‐dose group (*n* = 26), which was considered sufficient to assess the incidence of gastrointestinal symptoms and other safety outcomes.

The low‐dose (LD) (24 g/person/day, 0.4 g/kg bw/day) and high‐dose (HD) (36 g/person/day, 0.6 g/kg bw/day) were selected in this study based on previous human tolerance studies and regulatory guidelines (Han et al. 2018; Samyang Corporation 2017; GRAS Associates LLC (Agent for L&P Food Ingredient Co. Ltd) 2021). The low‐dose corresponds to the 90th percentile Estimated Daily Intake (EDI) of 0.42 g/kg body weight per day for U.S. individuals over 2 years old, as reported in GRN 693 (Samyang Corporation 2017), and aligns with Han et al.'s (2018) maximum single dose recommendation of 0.4 g/kg bw. This dosage is comparable to the peak cumulative EDI for adult males at 23.4 g/day within the same literature, thus remaining within a verified safe range. The high‐dose adheres to the maximum tolerated dose for females set at 0.6 g/kg bw per day according to GRN 693 and stays below Han et al.'s upper limit of 0.9 g/kg bw, representing a reasonable extension beyond the commercial safe dose of 30 g/day outlined in GRN 1029 (GRAS Associates LLC (Agent for L&P Food Ingredient Co. Ltd) 2021). Literature on animal toxicology indicates that *D*‐allulose has an LD_50_ value of 16.3 g/kg bw and a NOAEL of 5 g/kg bw per day from rat studies—values significantly higher than those used in this investigation (Matsuo et al. 2002; An et al. 2019). Additionally, incorporating *D*‐allulose into beverages at concentrations of 12%–18% falls well within the established range of 2%–100% for food additives. Thus, this dosing strategy is considered both safe and practical.

### 2.3 Participants

Participants were recruited via flyers, social media, and website advertisements. All participants provided written informed consent. Inclusion criteria were: adult men and women aged from 18 to 65 years old, with a body mass index within the normal range (18.5 ≤ BMI ≤ 24), and a weight ≥ 60 kg; and normal total cholesterol (TC) < 5.72 mmol/L, triglycerides (TG) < 1.70 mmol/L (the lipid levels of the participants were tested after fasting for 12–14 h) and normal blood glucose (fasting blood glucose < 7 mmol/L, or 2‐h postprandial blood glucose < 11.1 mmol/L, or glycated hemoglobin (HbA1c) < 6.5%). Exclusion criteria were: pregnant or breastfeeding; individuals with diabetes who are taking oral hypoglycemic drugs or insulin injections; hyperlipidemia; hypertension and taking diuretics; severe systemic diseases such as cardiovascular and cerebrovascular diseases, liver, kidney and hematopoietic system diseases; severe gastrointestinal diseases; gout or porphyria; mental diseases such as depression, schizophrenia; alcoholism or drug poisoning; history of surgery within 6 months; history of using functional foods that may affect the results of this study; history of cancer diagnosis and treatment; history of asthma or other allergies; history of alcohol consumption or regular smoking; other concurrent diseases that are being treated; unsuitable to participate in the clinical trial judged by the researchers.

### 2.4 Randomization and Masking

The randomization allocation sequence was generated by an investigator who was an independent researcher from a third‐party vendor. Participants who met the inclusion criteria and provided consent were randomized in nearly 1:1 ratio to an LD group or a HD group. Researchers, clinic study center staff, and participants remained blinded during the study period.

### 2.5 Procedures

This study was divided into a screening period, a intervention beverage period, and a follow‐up period. During the screening period, the subjects were informed and signed the informed consent form. Then, demographic data registration, medical history and medication history collection, and a questionnaire survey on daily dietary habits were completed.

Baseline examination: Before the examination, the subjects were required to fast overnight (starting at 20:00). The following items were conducted: assessment of the general condition of the subjects (including mental state and sleep quality). Physical examination (including weight, BMI, blood pressure, pulse rate, etc.); body composition analysis (including body fat, body fat percentage (BFP), muscle mass, bone mineral density (BMD), etc.), upper abdomen ultrasonography examination, and chest X‐ray examination; completion of electrocardiogram testing and vital sign measurement, collection of blood, urine and fecal samples. All blood samples were collected under standardized overnight fasting and consistent hydration conditions to minimize plasma volume variation. The samples were sent to the laboratory by a dedicated person for testing, including hematological examination, serum biochemical testing, urinalysis (including Urinary pH, number of red blood cells in urine (U‐RBC), number of white blood cells in urine (U‐WBC), number of epithelial cells in urine (U‐EC), etc.), and fecal analysis (including fecal appearance examination and microscopic examination). The subjects' eligibility for inclusion and exclusion criteria was determined based on the examination results.

Screening and enrollment: If the participants met the inclusion criteria and signed the informed consent voluntarily, they were randomly divided into two groups according to the order of enrollment: the high‐dose group and the low‐dose group. During the 30‐day intervention period, the high‐dose group subjects consumed the intervention beverage sample containing 18 g of *D*‐allulose per 100 mL (twice a day, with breakfast and dinner, with a total daily intake of 36 g per person per day), and the low‐dose group subjects consumed the intervention beverage sample containing 12 g of *D*‐allulose per 100 mL (twice a day, with breakfast and dinner, with a total daily intake of 24 g per person per day). Participants were requested to record short videos of daily intervention beverage intake using mobile phones, and adherence was monitored by research staff.

Participants were required to comply with the research center's supervision and complete all required records. All participants in the two groups completed the intervention beverage questionnaire survey through the WeChat Questionnaire Star mini‐program on their mobile phones every day. Video recording was not mandatory; however, missing records were documented and excluded from compliance analysis. The subjects did not need to change their original dietary and exercise habits during the intervention period.

Examination after 30 days of intervention beverage: After the intervention beverage study, the subjects returned to the hospital for safety follow‐up and repeated the baseline period's various examinations to assess the general condition of the subjects, including physical examination, body composition analysis, electrocardiogram testing and laboratory examination (including hematological examination, serum biochemical testing, urinalysis and fecal analysis). Subjects in the high‐dose group were required to provide fecal samples for gut microbiota analysis. Adverse events were also recorded. All examination processes were carried out by standard operating procedures, and the examination results were promptly recorded and archived.

### 2.6 Outcomes

The primary outcomes of the study were divided into two parts. One part of the outcomes consisted of the questionnaire survey results, which were primarily utilized to analyze the gastrointestinal tolerance of the subjects following the consumption of *D*‐allulose. This included GI symptoms such as nausea, bloating, colic, headache, satiety, decreased appetite and diarrhea. Additionally, it examined factors like the daily stool frequency and shape of the subjects during the trial period. The other primary outcomes included baseline and post‐intervention (Day 31) examination results from Peking University Third Hospital, encompassing hematological tests, serum biochemical assays, urinalysis, and fecal analysis, as well as body composition measurements and vital sign assessments.

Regarding laboratory tests, results of hematological examination parameters were obtained using a automated hematology analyzer (Sysmex XN1000, Japan). Serum biochemical parameters (including triglyceride (TG), high‐density lipoprotein cholesterol (HDL), low‐density lipoprotein cholesterol (LDL), etc.) were measured with a biochemical analyzer (Hitachi 7180, Japan). Urinalysis was analyzed by a automated urine analyzer (Sysmex UC3500, Japan), and fecal analysis was manually inspected (using test strips manufactured by ABON).

For physiological measurements, the main vital signs were monitored using a Mortara electrocardiograph (ELI380). Digital medical X‐ray imaging was performed with a United Imaging uDR 780i system. Medical ultrasound diagnoses were carried out using various devices, namely GE Vivid E9, GE Logiq S8, GE Logiq Fortis Express, Philips EPIQ 7C, and Rion Acclatix Lxp ECP. Physical examination metrics were evaluated with a body composition analyzer (InBody770), bone density was measured by an ultrasonic bone densitometer (BMD‐1000D). Compared with dual‐energy X‐ray absorptiometry (DXA), this ultrasonic method is radiation‐free, making it ethically preferable for healthy volunteer screening in a non‐clinical dietary intervention study. The instrument provides high reproducibility for population‐based observations, with a least significant change (LSC) of 0.1 for the T‐score, ensuring that the observed BMD changes are interpretable within measurement error range. Skin fold thickness was determined using a skin fold caliper (PZJ‐01).

### 2.7 Statistical Analysis

The research institution exercises internal quality management over the research process, data and biological sample collection, document archiving, and completion status. All raw data are stored in the Electronic Source Data Repository (ESDR) system of Peking University Third Hospital. Quality control (QC) is initiated concurrently with data entry, and the QC records are also archived in the ESDR system. For this study, an Electronic Data Capture (EDC) system was employed for data management.

Statistical analyses in this study were conducted using SPSS 22.0 software. All statistical tests were two‐tailed. For data conforming to a normal distribution (Shapiro–Wilk test) when comparing between the two dose groups, a *t*‐test was applied; for data not conforming to a normal distribution, a rank‐sum test was utilized. For categorical variables, a chi‐square test was used to compare the distribution differences between the high‐dose (HD) and low‐dose (LD) groups; when the expected frequency of any cell in the contingency table was < 5, Fisher's exact test was employed instead of the chi‐square test to ensure statistical validity. In addition, for continuous, normally distributed variables, to analyze the differences in these variables before (Day 0) and after the intervention (Day 31) among different groups, analysis of covariance (ANCOVA) was conducted with the baseline as a covariate. A significance level of *p* < 0.05 was adopted as the criterion for a statistically significant difference (except where otherwise specified). For continuous variables, the sample size, mean, standard deviation, median, minimum, and maximum values were presented. For categorical variables, their frequencies and percentages were presented in tabular form. Chi‐square test was used to compare GI symptom incidence between groups.

## 3 Results

### 3.1 Baseline Demographics and Clinical Characteristics of Participants

**FIGURE 1:**
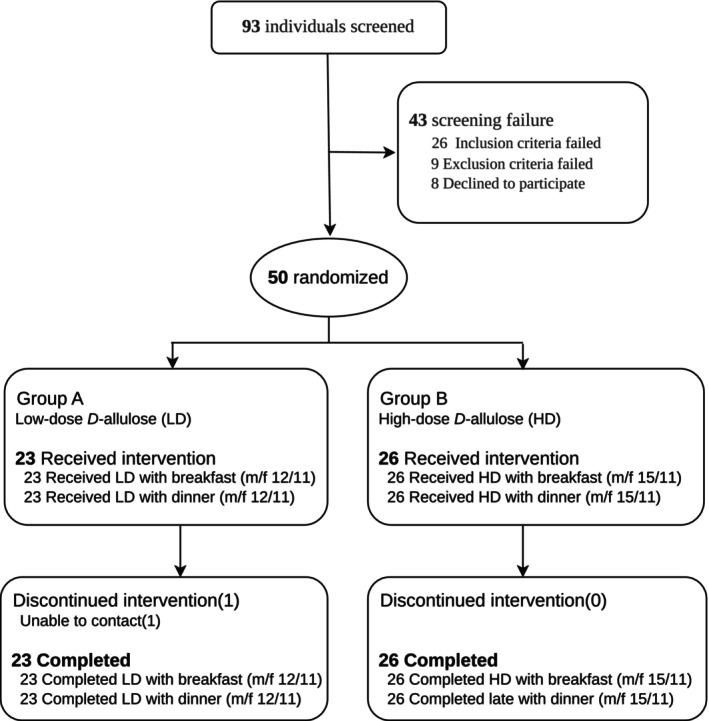
The profile of the study. A total of 93 individuals were screened for this trial. Subsequently, 50 participants were randomly assigned in a nearly 1:1 ratio to receive intervention with LD (Group A, *n* = 24) or HD (Group B, *n* = 26). Following the dropout of one individual from Group A, a final cohort of 49 participants remained in the study (Group A, *n* = 23 and Group B, *n* = 26). Each participant was permitted to take the sample twice daily with breakfast and dinner over a one‐month trial period. At the end of the trial, 23 participants completed the LD intervention (male/female: 12/11), while 26 participants completed the HD intervention (male/female: 15/11).

October 24 to November 1, 2024, 93 individuals were screened for this trial, with 43 being excluded because they did not meet the inclusion criteria, met the exclusion criteria, or declined to participate. A total of 50 participants were randomly assigned nearly 1:1 to receive intervention with LD (group A, *n* = 24) or HD (group B, *n* = 26). After one individual (group A, *n* = 1) dropped out, 49 individuals (group A, *n* = 23; group B, *n* = 26) finally participated in the study. The dropout was due to loss of contact with the participant. During one‐month trial period, each participant was instructed to consume the intervention beverage twice daily: once with breakfast (from 6 a.m. to 2 p.m.) and once with dinner (from 4 p.m. to 11 p.m.), which was supervised and recorded by the research staff. At the end of the study, 23 participants (male/female 12/11) completed the LD intervention and 26 participants (male/female 15/11) completed the HD intervention (Figure 1).

The demographic characteristics and specific medical examination indicator profiles of participants were generally comparable between the HD and LD groups, including age, gender, ethnicity, height, weight, BMI, T‐βHCG (only female), upper abdomen ultrasonography, and chest X‐ray results (Table 1 and Table S2).

**TABLE 1:** Baseline demographics and physical characteristics of participants.

	HD group (*n* = 26)	LD group (*n* = 24)	Total (*n* = 50)
Age (years)			
Mean ± SD	38.6 ± 11.47	32.0 ± 10.33	35.4 ± 11.33
Gender, *n* (%)			
Male	15 (57.7)	13 (54.2)	28 (56.0)
Female	11 (42.3)	11 (45.8)	22 (44.0)
Ethnicity, *n* (%)			
Han	24 (92.3)	23 (95.8)	47 (94.0)
Not Han	2 (7.7)	1 (4.2)	3 (6.0)
Height (cm)			
Mean ± SD	169.2 ± 5.39	168.3 ± 4.97	168.8 ± 5.16
Weight (kg)			
Mean ± SD	66.1 ± 4.91	64.1 ± 2.82	65.1 ± 4.13
BMI^a^ (kg/m^2^)			
Mean ± SD	23.08 ± 0.818	22.64 ± 1.122	22.87 ± 0.991

^a^ BMI, body mass index.

### 3.2 Mild, Transient Gastrointestinal Symptoms With No Dose‐Dependent Differences

It should be noted that although one participant was lost to follow‐up during the intervention period, the questionnaire data collected prior to his/her withdrawal were still incorporated into the study's analytical dataset in accordance with the predefined data inclusion criteria. This study collected a total of 1388 self‐reported questionnaires on gastrointestinal symptoms after intervention beverage consumption, with 740 from the high‐dose group and 648 from the low‐dose group during 30‐day intervention. These questionnaires were designed to assess the gastrointestinal (GI) symptoms and defecation conditions of participants after taking the test samples. The gastrointestinal (GI) symptoms included nausea, bloating, colic, headache, satiety, decreased appetite and diarrhea. The defecation conditions mainly referred to stool frequency.

To improve interpretability, we explicitly distinguished between two metrics: questionnaire‐level frequency (the percentage of all 1388 questionnaires in which a given symptom was reported) and participant‐level incidence (the percentage of participants who reported a given symptom at least once during the 30‐day intervention).

**FIGURE 2:**
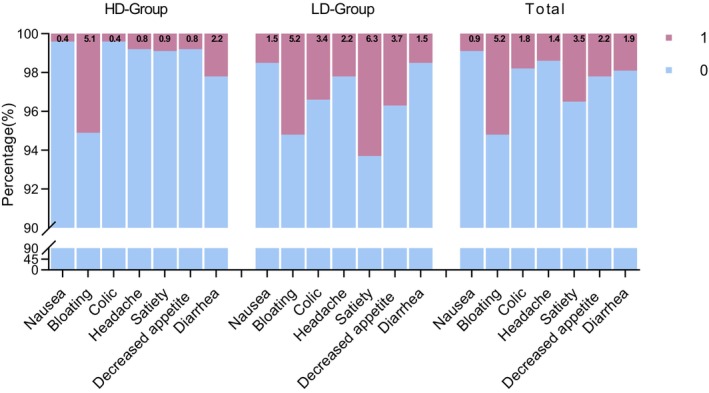
Questionnaire‐level frequency of gastrointestinal (GI) symptoms during the 30‐day *D*‐allulose intervention. Data are shown as the percentage of all 1388 completed questionnaires in which a given symptom was reported. Results are presented separately for the high‐dose (HD) group, low‐dose (LD) group, and overall participants (all subjects combined). For each of the seven symptoms (nausea, bloating, colic, headache, satiety, decreased appetite, and diarrhea), the stacked bar chart shows: Blue segments for asymptomatic responses (grade 0) and red segments for symptomatic responses (grades 1–3). For example, the frequency of nausea was 0.4% in the HD group, 1.5% in the LD group, and 0.9% overall.

Questionnaire‐level frequency of GI symptoms reported in the questionnaires during the 30‐day *D*‐allulose intervention was analyzed (Figure 2). The results showed that the frequency of nausea was 0.9% (HD group, 0.4%; LD group, 1.5%); the frequency of bloating was 5.2% (HD group, 5.1%; LD group, 5.2%); the frequency of colic was 1.8% (HD group, 0.4%; LD group, 3.4%); the frequency of headache symptoms was 1.4% (HD group, 0.8%; LD group, 2.2%); the frequency of satiety was 3.5% (HD group, 0.9%; LD group, 6.3%); the frequency of decreased appetite was 2.2% (HD group, 0.8%; LD group, 3.7%); and the frequency of diarrhea was 1.9% (HD group, 2.2%; LD group, 1.5%) (Figure 2).

**FIGURE 3:**
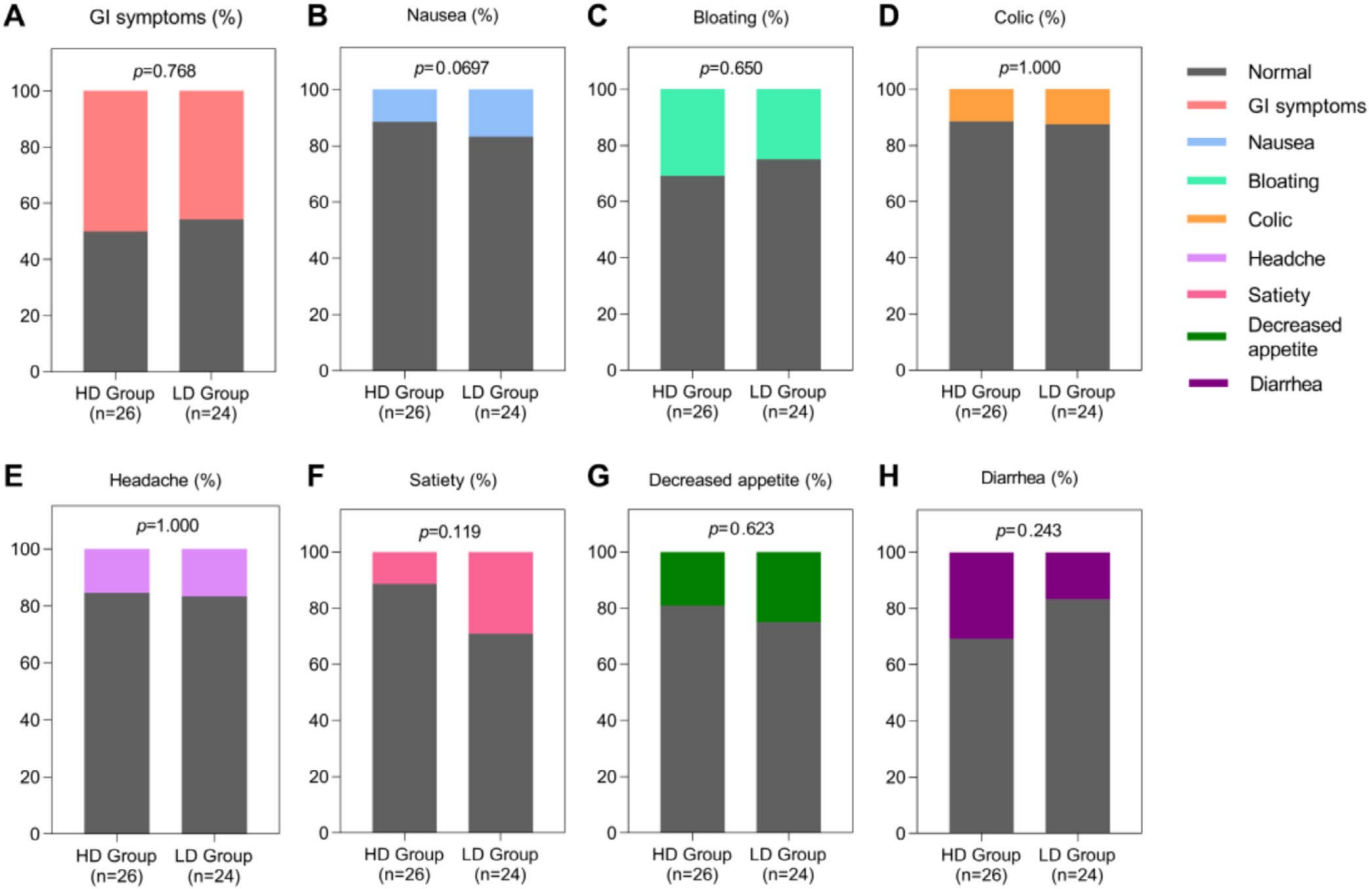
Participant‐level incidence of GI symptoms in the HD and LD groups during the 30‐day intervention (percentage of participants reporting a symptom at least once). This set of stacked bar charts (A–H) shows the percentage of participants with normal status (gray segment) versus symptomatic status (colored segments) for overall GI symptoms and individual symptoms, within the HD group (*n* = 26) and LD group (*n* = 24): Panel A displays overall GI symptom prevalence: 50.0% in the HD group compared to 45.8% in the LD group, with no significant difference between groups (*p* = 0.768). Panels B–H detail individual symptoms: Nausea (B, light blue), bloating (C, teal), colic (D, orange), headache (E, purple), satiety (F, pink), decreased appetite (G, green), and diarrhea (H, dark purple). The gray segment indicates participants without unusual symptoms; all reported *p*‐values show no significant differences between HD and LD groups (all *p* > 0.05).

Participant‐level incidence based on the results of the 30‐day questionnaire survey, the number of participants in both groups who reported GI symptoms after taking the test samples was analyzed (Figure 3). During the test period, a total of 24 participants (48.0%) reported GI symptoms using the questionnaire, with 13 (50.0%) in the high‐dose group and 11 (45.8%) in the low‐dose group. There was no statistically significant difference between the two dose groups (*p* > 0.05) (Figure 3). When it comes to each specific GI symptom, there were 7 cases (14.0%) of nausea, including 3 cases (11.5%) in the high‐dose group and 4 cases (16.7%) in the low‐dose group (*p* > 0.05); 14 cases (28.0%) of bloating, including 8 cases (30.8%) in the high‐dose group and 6 cases (25.0%) in the low‐dose group (*p* > 0.05); 6 cases (12.0%) of colic, including 3 cases (11.5%) in the high‐dose group and 3 cases (12.5%) in the low‐dose group (*p* > 0.05); 8 cases (16.0%) of headache, including 4 cases (15.4%) in the high‐dose group and 4 cases (16.7%) in the low‐dose group (*p* > 0.05); 10 cases (20.0%) of satiety, including 3 cases (11.5%) in the high‐dose group and 7 cases (29.2%) in the low‐dose group (*p* > 0.05); decreased appetite was observed in 11 cases (22.0%), with 5 cases (19.2%) in the high‐dose group and 6 cases (25.0%) in the low‐dose group (*p* > 0.05); diarrhea was observed in 12 cases (24.0%), including 8 cases (30.8%) in the high‐dose group and 4 cases (16.7%) in the low‐dose group (*p* > 0.05). There was no statistically significant difference between the two dose groups in each specific GI symptom (*p* > 0.05) (Figure 3).

**FIGURE 4:**
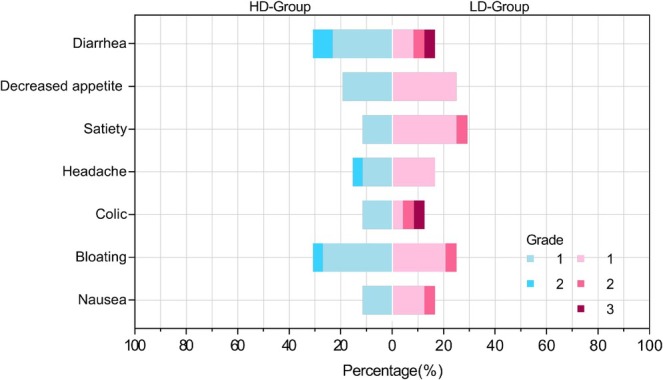
Distribution of severity grades for gastrointestinal (GI) symptoms in high‐dose (HD) and low‐dose (LD) groups. This figure shows the percentage distribution of severity grades (1–3) for seven gastrointestinal symptoms—nausea, bloating, colic, headache, satiety, decreased appetite, and diarrhea—in the HD and LD groups. The x‐axis indicates percentages with the midpoint (0) separating the HD group on the left from the LD group on the right; the y‐axis lists symptom types. Colored segments represent severity grades as follows: Grade 1 (light cyan for HD, light pink for LD); Grade 2 (cyan for HD, red for LD); Grade 3 (purple for LD). Most symptomatic participants reported grade 1 symptoms—the most common severity level—while only a small number experienced grade 2 or 3 symptoms.

In this questionnaire, the various gastrointestinal reactions were graded from 0 to 3, with grade 0 being normal (no unusual symptoms), and the higher the grade, the more severe the symptoms. The percentage distribution of severity grades (1–3) for seven gastrointestinal symptoms—nausea, bloating, colic, headache, satiety, decreased appetite, and diarrhea—was analyzed in the HD and LD groups. The results showed that the majority of GI symptoms were at grade 1, with only a few subjects reporting grade 2 or 3. No statistically significant differences in symptom severity distribution were observed between the two dose groups (all *p* > 0.05) (Figure 4).

### 3.3 Transient, Low‐Incidence Abnormal Bowel Movements Across D‐Allulose Dose Groups

**FIGURE 5:**
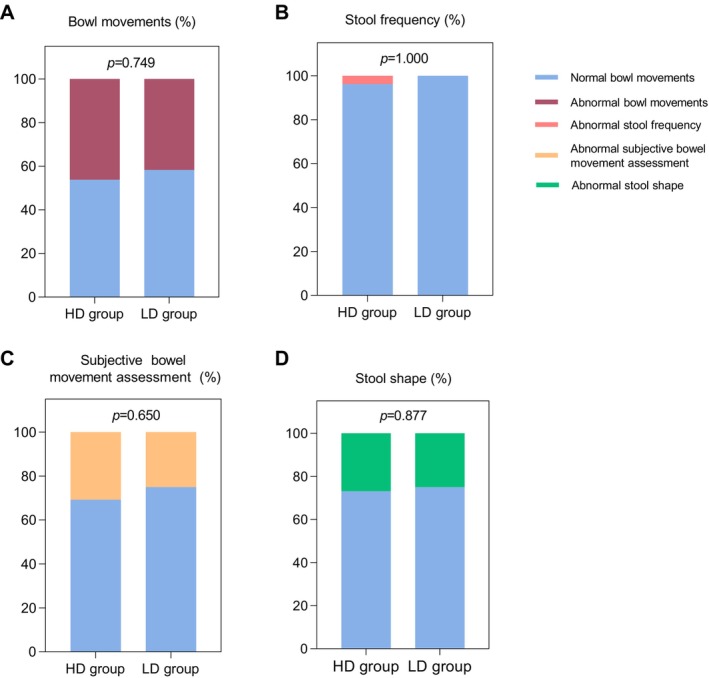
Normal vs. abnormal defecation outcomes in the high‐dose (HD) and low‐dose (LD) groups. This panel of stacked bar charts (A–D) displays the percentage of participants with normal (blue segments) or abnormal (colored segments) status across four defecation indices: (A) Bowel movements (including stool frequency, subjective bowel movement assessment and stool shape) (abnormal = dark red; *p* = 0.749); (B) Stool frequency (abnormal = pink; only rare cases in HD group; *p* = 1.000); (C) Subjective bowel movement assessment (abnormal = orange; *p* = 0.650); (D) Stool shape (abnormal = green; *p* = 0.877).

**FIGURE 6:**
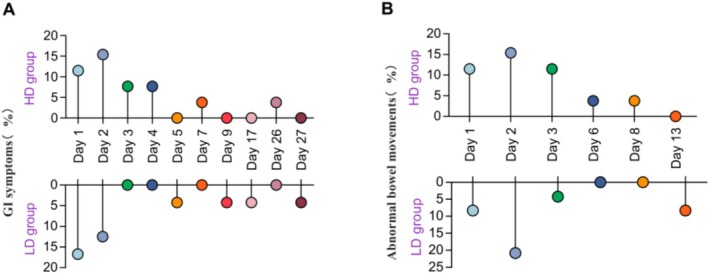
Temporal dynamics of GI symptoms and abnormal bowel movements in high‐dose (HD) and low‐dose (LD) groups after consuming *D*‐allulose beverages. (A) Proportion of participants with GI symptoms on specific days; the upper panel shows the HD group, while the lower panel shows the LD group. (B) Proportion of participants with abnormal bowel movements on distinct days; the upper panel represents the HD group, and the lower panel reflects the LD group.

In this study, we analyzed and compared the percentages of participants who had aberrant defecation responses across the two dose groups. The results showed that a total of 22 participants (44.0%) reported abnormal bowel movements (including abnormal stool frequency, subjective abnormal bowel movement and abnormal stool shapes) during the trial period, with 12 subjects (46.2%) in the high‐dose group and 10 subjects (41.7%) in the low‐dose group (Figure 5). There were no statistically significant differences between the two dose groups (*p* > 0.05, Figure 5A). Notably, only one subject (2.0%) in the high‐dose group had abnormal stool frequency (abnormal stool frequency was defined as > 3 bowel movements per day or < 1 per 2 days). 14 subjects (28.0%) reported subjective abnormal bowel movement (*p* > 0.05), 20.0% of participants with subjective bowel discomfort reported a sensation of dragging; 13 subjects (26.0%) exhibited abnormal stool shapes (*p* > 0.05), predominantly loose fragments (30.0%) or pasty/watery stools (26.0%); There was no statistically significant difference in each defecation index between the two dose groups (*p* > 0.05, Figure 5B–D). In addition, this study conducted a statistical analysis on the number of days with GI symptoms and abnormal bowel movements. This index showed no statistically significant difference in this index between the two dose groups (*p* > 0.05). GI symptoms (Figure 6A) and abnormal bowel movements (Figure 6B) were mostly observed between Days 1 and 3 after consuming test drinks containing *D*‐allulose (Figure 6).

### 3.4 No Clinically Significant Abnormalities in Routine Laboratory Indicators

Blood, urine, and fecal samples were collected from participants on day 0 and after 30 days of the intervention period. These samples were used for hematological examination, serum biochemical testing, urinalysis (including urinary pH, red blood cells (U‐RBC), white blood cells (U‐WBC), and epithelial cells (U‐EC) in urine), and fecal analysis (including fecal appearance and microscopic examination). All laboratory results were reviewed and interpreted by licensed physicians. The results indicated that the majority of indicators for both groups of participants remained within the normal range. No clinically significant (CS) abnormalities were detected in either group. All abnormalities were considered non‐clinically significant (NCS).

### 3.5 Stable Vital Signs and Body Composition (Except for High‐Dose BMD T‐Score Shifts)

For the subjects in both dose groups, no significant changes were observed in vital signs after consuming the test food compared to baseline. These vital signs included systolic blood pressure (SBP), diastolic blood pressure (DBP), and pulse rate (PR). Additionally, most physical examination indicators—such as weight, body mass index (BMI), skin fold thickness, BMD Z‐score (for subjects under 35 years old), body fat, muscle mass, skeletal muscle, body fat percentage (BFP), and basal metabolic rate (BMR)—showed no significant changes (all *p* > 0.05) (Table 2). Furthermore, apart from BMD T‐score, no significant differences were observed between the two dose groups when comparing the changes in these indicators before and after consuming the test food (all *p* > 0.05) (Table 3).

**TABLE 2:** Vital signs and physical examination parameters of participants before and after intervention.

	HD group		*p*	LD group		*p*
	Baseline (*n* = 26)	Day 31 (*n* = 26)		Baseline (*n* = 24)	Day 31 (*n* = 23)^a^	
SBP (mmHg)	119.0 ± 10.70	117.7 ± 11.18	0.435	117.2 ± 8.57	119.8 ± 8.91	0.233
DBP (mmHg)	71.7 ± 7.28	71.6 ± 6.87	0.930	69.6 ± 7.57	70.3 ± 7.31	0.791
PR (beats/min)	70.8 ± 11.52	66.7 ± 9.36	0.067	67.8 ± 7.31	68.8 ± 10.65	0.419
Weight (kg)	66.11 ± 4.911	66.52 ± 5.372	0.141	64.08 ± 2.823	64.33 ± 3.469	0.724
BMI (kg/m^2^)	23.08 ± 0.818	23.23 ± 1.026	0.139	22.64 ± 1.122	22.77 ± 1.125	0.738
Skinfold thickness (mm)	2.902 ± 1.1019	2.823 ± 1.1404	0.328	2.752 ± 0.7606	2.767 ± 0.7859	0.508
Body fat	18.58 ± 4.278	18.92 ± 4.050	0.276	16.80 ± 4.521	17.36 ± 4.772	0.165
Muscle mass	45.16 ± 6.804	44.87 ± 6.762	0.306	44.54 ± 5.506	44.33 ± 6.045	0.532
Skeletal muscle	26.24 ± 4.255	26.09 ± 4.251	0.377	26.04 ± 3.645	25.94 ± 3.888	0.673
BFP	28.18 ± 7.025	28.33 ± 7.131	0.773	26.35 ± 7.366	27.11 ± 7.841	0.191
BMR	1403.2 ± 152.97	1397.1 ± 152.66	0.335	1388.9 ± 123.72	1384.6 ± 136.01	0.576
BMD T‐score	0.157 ± 0.7138 (*n* = 16)	−0.197 ± 0.6543 (*n* = 16)	0.043	−0.322 ± 0.4195 (*n* = 6)	0.175 ± 0.6722 (*n* = 6)	0.067
BMD Z‐score	0.369 ± 0.5133 (*n* = 10)	0.315 ± 0.5155 (*n* = 10)	0.685	0.111 ± 0.5258 (*n* = 18)	0.145 ± 0.5157 (*n* = 17)	0.728

*Note:* *p* values were calculated by paired *t*‐test for intra‐group comparison (baseline vs Day 31). Underlined *p* < 0.05 denotes a statistically significant difference.

Abbreviations: BFP, body fat percentage; BMD, bone mineral density; BMI, body mass index; BMR, basal metabolic rate; DBP, diastolic blood pressure; PR, pulse rat; SBP, systolic blood pressure.

^a^ (*n* = 23), one participant in Group A was lost to follow‐up, resulting in a final analyzed cohort of 49 participants.

**TABLE 3:** Between‐group differences in changes of vital signs and body composition post‐intervention.

	HD group (*n* = 26)	LD group (*n* = 23)	*p*
Weight (kg)	0.41 ± 1.384	0.10 ± 1.303	0.684
BMI (kg/m^2^)	0.15 ± 0.489	0.03 ± 0.438	0.473
Skinfold thickness (mm)	−0.079 ± 0.4070	−0.052 ± 0.3776	0.831
Body fat	0.34 ± 1.562	0.36 ± 1.214	0.895
Muscle mass	−0.29 ± 1.421	−0.18 ± 1.401	0.797
Skeletal muscle	−0.15 ± 0.860	−0.08 ± 0.893	0.783
BFP	0.15 ± 2.597	0.50 ± 1.792	0.612
BMR	−6.1 ± 32.09	−3.7 ± 32.08	0.797
BMD T‐score (Age)	−0.354 ± 0.6293 (*n* = 16)	0.497 ± 0.4963 (*n* = 6)	0.030
BMD Z‐score	−0.05 ± 0.425 (*n* = 10)	0.05 ± 0.602 (*n* = 17)	0.806

*Note:* *p* values were calculated by analysis of covariance (ANCOVA) to compare the changes in each index (Day 31 minus baseline) between groups after adjusting for baseline values. Underlined *p* < 0.05 denotes a statistically significant difference.

Abbreviations: BFP, body fat percentage; BMD, bone mineral density; BMI, body mass index; BMR, basal metabolic rate.

### 3.6 Adaptive Hematological Changes Without Toxicological Significance

As stated above, no CS were detected among participants. No participant approached the lower limits of normal ranges for RBC, HCT, or PLT according to our hospital laboratory's reference values. To minimize plasma volume variation, all blood samples were collected under standardized overnight fasting conditions with consistent hydration instructions (free access to plain water).

**FIGURE 7:**
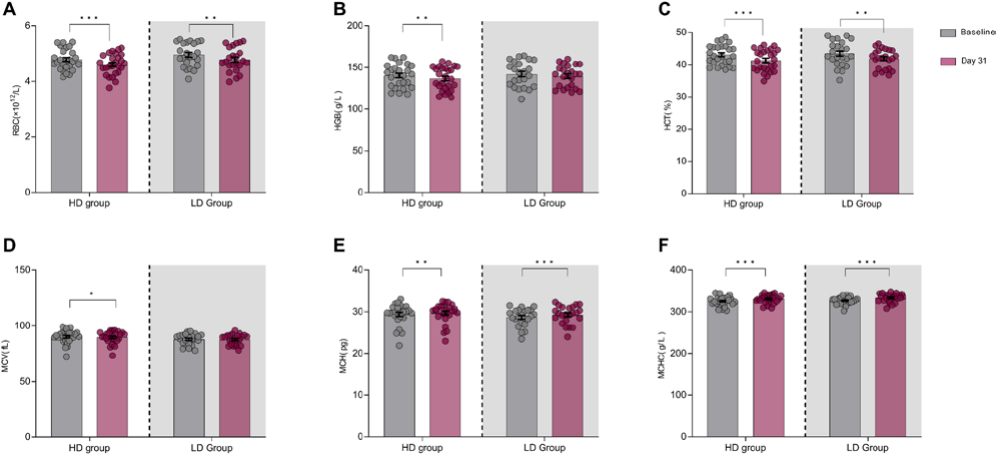
Changes in hematological parameters in high‐dose (HD) and low‐dose (LD) groups at baseline and on day 31 post‐intervention. Panels A–F show the distributions of red blood cell count (RBC, A), hemoglobin level (HGB, B), hematocrit (HCT, C), mean corpuscular volume (MCV, D), mean corpuscular hemoglobin (MCH, E), and mean corpuscular hemoglobin concentration (MCHC, F). Gray dots represent baseline samples; rose red dots indicate samples from day 31. Each dot represents an individual participant (HD group, *n* = 26; LD group, *n* = 23). Change from baseline is presented as mean ± standard error of the mean (SEM). *p < 0.05, **p < 0.01, ***p < 0.001: Significant differences compared to baseline in each group (paired *t* test).

**FIGURE 8:**
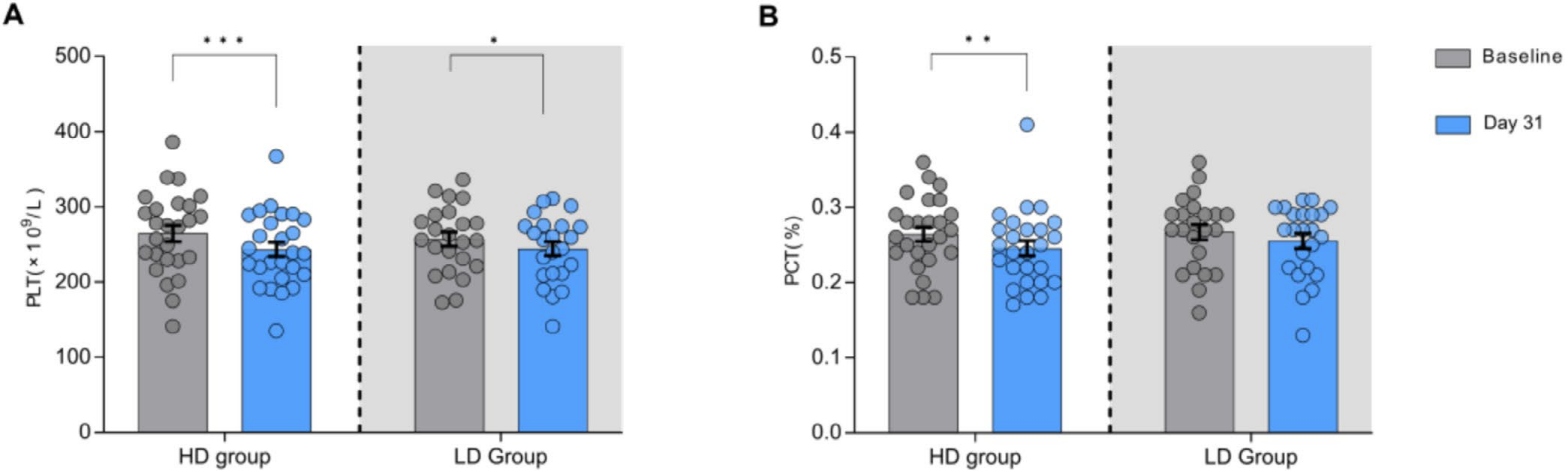
Changes in platelet parameters in high‐dose (HD) and low‐dose (LD) groups at baseline and after a 30‐day intervention (A, B) Show the levels of platelet count (PLT) (A) and plateletcrit (PCT) (B) for both groups at baseline and post‐intervention. Gray dots represent baseline samples; blue dots indicate samples from day 31. Each dot represents an individual participant (HD group, *n* = 26; LD group, *n* = 23). Data are presented as mean ± standard error of the mean (SEM). Statistical significance compared to baseline is indicated as follows: *p < 0.05, **p < 0.01, ***p < 0.001 (paired *t* test).

After the 30‐day *D*‐allulose intervention, however, both groups exhibited significant differences in some hematological parameters compared to their baselines. Figure 7 and Table 4 demonstrate that, when compared with baseline, RBC (HD group, *p* < 0.001; LD group, *p* = 0.007, Figure 7A) and HCT (HD group, *p* < 0.001; LD group, *p* = 0.005, Figure 7C) decreased significantly in both the HD and LD groups. Additionally, significant increases in mean hemoglobin (MCH) (HD group, *p* = 0.004; LD group, *p* < 0.001, Figure 7E) and mean corpuscular hemoglobin concentration (MCHC) (both groups, *p* < 0.001, Figure 7F) were observed after 30 days of intervention compared to baseline. Only the HD group showed a significant decrease in the mean corpuscular volume (MCV) (*p* < 0.05, Figure 7D) and hemoglobin level (HGB) (*p* < 0.01, Figure 7B). In the platelet parameters, there was a notable reduction in platelet count (PLT) for both groups (HD group, *p* < 0.001; LD group *p* = 0.023, Figure 8A). However, a significant decrease in plateletcrit (PCT) was observed only in the HD group (*p* = 0.004, Figure 8B). Other parameters, such as white blood cell count (WBC), neutrophil percentage (NEUT%), monocyte percentage (MONO%), and eosinophil percentage (EO%), showed no statistically significant differences from the baseline (*p* > 0.05) (Table 4).

**TABLE 4:** Hematological, serum biochemical, and urinalysis parameters at baseline and post‐intervention.

	HD group		*p*	LD group		*p*
	Baseline (*n* = 26)	Day 31 (*n* = 26)		Baseline (*n* = 24)	Day 31 (*n* = 23)	
WBC (×10^9^/L)	6.068 ± 1.4646	5.673 ± 1.3540	0.164	6.121 ± 1.0008	5.584 ± 1.3427	0.105
RBC (×10^12^/L)	4.780 ± 0.3756	4.605 ± 0.3734	< 0.001	4.960 ± 0.4175	4.780 ± 0.4298	0.007
HGB (g/L)	140.2 ± 13.81	136.7 ± 13.83	0.007	142.6 ± 15.13	139.6 ± 13.01	0.152
HCT (%)	43.05 ± 3.092	41.27 ± 3.350	< 0.001	43.56 ± 3.819	41.81 ± 3.015	0.005
MCV (fL)	90.30 ± 5.982	89.78 ± 5.470	0.043	87.94 ± 4.889	87.70 ± 4.644	0.751
MCH (pg)	29.40 ± 2.522	29.72 ± 2.363	0.004	28.77 ± 2.104	29.27 ± 2.146	< 0.001
MCHC (g/L)	325.2 ± 11.35	330.8 ± 10.15	< 0.001	327.0 ± 8.99	333.6 ± 10.24	< 0.001
PLT (×10^9^/L)	264.6 ± 55.05	243.2 ± 48.71	< 0.001	256.6 ± 43.63	244.2 ± 44.53	0.023
PCT (%)	0.264 ± 0.0488	0.245 ± 0.0512	0.004	0.266 ± 0.0480	0.255 ± 0.0478	0.069
NEUT%	58.06 ± 7.478	60.69 ± 8.685	0.233	58.24 ± 8.544	60.03 ± 8.250	0.591
NEUT#	3.573 ± 1.1283	3.494 ± 1.1889	0.783	3.598 ± 0.9160	3.387 ± 1.1378	0.361
LYMPH%	32.09 ± 6.313	29.01 ± 7.781	0.101	32.06 ± 6.952	29.70 ± 6.710	0.239
MONO%	7.41 ± 1.985	7.90 ± 1.669	0.188	7.00 ± 1.564	7.23 ± 1.915	0.306
EO%	1.83 ± 1.082	1.79 ± 1.006	0.811	2.15 ± 1.283	2.44 ± 1.840	0.303
ALT (U/L)	14.1 ± 4.90	13.7 ± 4.55	0.594	13.5 ± 4.95	12.5 ± 3.79	0.277
AST (U/L)	18.5 ± 2.72	18.6 ± 5.40	0.938	17.3 ± 2.68	16.0 ± 2.64	0.054
TBIL (μmol/L)	9.603 ± 3.6125	10.733 ± 3.4030	0.203	11.366 ± 3.4078	9.430 ± 3.3187	0.035
ALP (U/L)	61.70 ± 14.854	52.85 ± 14.554	< 0.001	61.56 ± 10.636	53.03 ± 10.265	< 0.001
γ‐GT (U/L)	16.6 ± 8.34	12.2 ± 5.98	< 0.001	17.0 ± 11.75	13.3 ± 7.05	0.015
CK (U/L)	102.8 ± 55.62	97.7 ± 43.39	0.654	107.3 ± 69.85	109.1 ± 58.22	0.894
Cr(μmol/L)	66.6 ± 10.83	67.2 ± 10.12	0.734	67.9 ± 14.73	66.1 ± 11.34	0.511
UA (μmol/L)	292.6 ± 68.15	260.7 ± 54.55	0.003	316.0 ± 65.22	285.5 ± 64.69	0.007
Urea (mmol/L)	4.790 ± 1.0515	4.880 ± 1.1558	0.687	4.398 ± 0.8969	4.745 ± 1.5137	0.102
TC (mmol/L)	4.588 ± 0.5318	4.326 ± 0.6966	0.015	4.548 ± 0.6281	4.269 ± 0.5889	0.030
TG(mmol/L)	0.802 ± 0.3001	0.828 ± 0.2881	0.668	0.898 ± 0.2658	0.997 ± 0.3842	0.431
HDL‐C (mmol/L)	1.439 ± 0.1626	1.183 ± 0.2044	< 0.001	1.403 ± 0.2011	1.188 ± 0.1890	< 0.001
LDL‐C (mmol/L)	2.703 ± 0.5447	2.760 ± 0.5679	0.555	2.670 ± 0.5439	2.659 ± 0.5492	0.868
FBG (mmol/L)	5.269 ± 0.3394	5.030 ± 0.2975	0.001	5.141 ± 0.3305	5.110 ± 0.4449	0.524
U‐SG	1.013 ± 0.0069	1.017 ± 0.0087	0.022	1.016 ± 0.0090	1.018 ± 0.0100	0.245
U‐pH	6.15 ± 0.745	6.13 ± 0.819	0.908	6.06 ± 0.727	6.11 ± 0.706	0.826
U‐RBC	5.319 ± 9.9749	4.181 ± 4.1120	0.534	3.592 ± 5.0586	3.787 ± 5.5976	0.363
U‐WBC	6.07 ± 12.788	8.47 ± 11.371	0.330	10.65 ± 25.408	19.63 ± 30.732	0.232
U‐EC	4.98 ± 8.911	5.18 ± 7.969	0.917	6.47 ± 8.617	13.72 ± 23.781	0.099

*Note:* *p* values were calculated by paired *t*‐test for intra‐group comparison (baseline vs Day 31). Underlined *p* < 0.05 denotes a statistically significant difference.

Abbreviations: WBC, white blood cell count; RBC, red blood cell Count; HGB, hemoglobin; HCT, hematocrit; MCV, mean corpuscular volume; MCH, mean corpuscular hemoglobin; MCHC, mean corpuscular hemoglobin concentration; PLT, platelet count; PCT, plateletcrit; NEUT%, neutrophil percentage; NEUT#, neutrophil; LYMPH%, lymphocyte percentage; MONO%, monocyte percentage; EO%, eosinophil percentage; ALT, alanine aminotransferase; AST, aspartate aminotransferase; TBIL, total bilirubin; ALP, alkaline phosphatase; γ‐GT, gamma‐glutamyl transferase; CK, creatine kinase; Cr, creatinine; UA, uric acid; TC, total cholesterol; TG, triglycerides; HDL‐C, high‐density lipoprotein; LDL‐C, low‐density lipoprotein; FBG, fasting blood glucose; U‐SG, urine specific gravity; U‐pH, urine pH value; U‐RBC, number of red blood cells in urine; U‐WBC, number of white blood cells in urine; U‐EC, number of epithelial cells in urine.

The two groups were compared in terms of the changes in hematological examination parameters from baseline (Day 0) to Day 31 (post intervention). The results showed that the alterations in all hematological examination indicators were not statistically significant (*p* > 0.05) (Table 5). Although there was no statistical significance, in terms of the trend of change, the white blood cell count (WBC) decreased in both groups (HD group, −0.395 ± 1.4043 × 10^9^/L; LD group, −0.575 ± 1.6308 × 10^9^ /L; *p* = 0.744). Among the parameters related to the red blood cell system, the red blood cell count (RBC) showed a downward trend in both groups (HD group, −0.175 ± 0.2321 × 10^12^/L; LD group, −0.171 ± 0.2747 × 10^12^/L; *p* = 0.599). The hemoglobin (HGB) levels also decreased in both groups (HD group, −3.5 ± 6.19 g/L, LD group, −2.3 ± 7.60 g/L, *p* = 0.420). The hematocrit (HCT) showed a similar decrease in both groups (HD group, −1.78% ± 1.915%; LD group, −1.60% ± 2.441%; *p* = 0.628). Conversely, the mean corpuscular hemoglobin (MCH) (HD group, 0.32 ± 0.524; LD group, 0.57 ± 0.398; *p* = 0.114) showed a slight increase. The mean corpuscular hemoglobin concentration (MCHC) also increased marginally in both the HD and LD groups (HD group, 5.6 ± 6.17; LD group, 6.8 ± 4.59; *p* = 0.344). The platelet count (PLT) decreased in both groups (HD group, −21.346 ± 28.0213 × 10^9^/L; LD group, −12.696 ± 24.8896 × 109/L; *p* = 0.325) (Table 5). The comparison of changes in parameters, such as the red blood cell system indicators discussed above in two groups from baseline to Day 31 correction indicated that the two dosages had similar impacts on these indicators.

**TABLE 5:** Between‐group differences in changes of hematological and biochemical indexes after 30‐day intervention.

	HD group (*N* = 26)	LD group (*N* = 23)	*p*
WBC (×10^9^/L)	−0.395 ± 1.4043	−0.575 ± 1.6308	0.744
RBC (×10^12^/L)	−0.175 ± 0.2321	−0.171 ± 0.2747	0.599
HGB (g/L)	−3.5 ± 6.19	−2.3 ± 7.60	0.420
HCT (%)	−1.78 ± 1.915	−1.60 ± 2.441	0.628
MCV (fL)	−0.52 ± 1.234	−0.10 ± 1.491	0.673
MCH (pg)	0.32 ± 0.524	0.57 ± 0.398	0.114
MCHC (g/L)	5.6 ± 6.17	6.8 ± 4.59	0.344
PLT (×10^9^/L)	−21.346 ± 28.0213	−12.696 ± 24.8896	0.325
PCT (%)	−0.019 ± 0.0305	−0.012 ± 0.0295	0.368
NEUT%	2.63 ± 10.985	1.02 ± 8.986	0.712
NEUT#	−0.078 ± 1.4348	−0.274 ± 1.4112	0.714
LYMPH%	−3.08 ± 9.232	−1.82 ± 7.214	0.676
MONO%	0.49 ± 1.841	0.32 ± 1.451	0.375
EO%	−0.04 ± 0.812	0.42 ± 1.897	0.615
ALT (U/L)	−0.4 ± 4.05	−1.0 ± 4.54	0.359
AST (U/L)	0.1 ± 5.10	−1.3 ± 3.05	0.117
TBIL (μmol/L)	1.130 ± 4.4341	−1.624 ± 3.4167	0.074
ALP (U/L)	−8.85 ± 9.475	−9.20 ± 9.497	0.938
γ‐GT (U/L)	−4.4 ± 3.99	−3.7 ± 6.64	0.358
CK (U/L)	−5.1 ± 58.26	2.0 ± 70.87	0.461
Cr(μmol/L)	0.6 ± 8.70	−1.2 ± 8.88	0.474
UA (μmol/L)	−31.9 ± 47.00	−30.1 ± 46.51	0.408
Urea (mmol/L)	0.090 ± 1.1474	0.427 ± 1.2024	0.549
TC (mmol/L)	−0.262 ± 0.5021	−0.265 ± 0.5406	0.898
TG(mmol/L)	0.027 ± 0.3158	0.086 ± 0.5218	0.112
HDL‐C (mmol/L)	−0.256 ± 0.1676	−0.224 ± 0.1496	0.591
LDL‐C (mmol/L)	0.057 ± 0.4889	0.012 ± 0.3533	0.606
FBG (mmol/L)	−0.239 ± 0.3119	−0.057 ± 0.4248	0.180
U‐SG	0.0040 ± 0.0083	0.0025 ± 0.0102	0.848
U‐pH	−0.02 ± 0.854	0.04 ± 0.952	0.998

*Note:* *p* values were calculated by analysis of covariance (ANCOVA) to compare the changes in each index (Day 31 minus baseline) between groups after adjusting for baseline values.

Abbreviations: ALP, alkaline phosphatase; ALT, alanine aminotransferase; AST, aspartate aminotransferase; CK, creatine kinase; Cr, creatinine; EO%, eosinophil percentage; FBG, fasting blood glucose; HCT, hematocrit; HDL‐C, high‐density lipoprotein; HGB, hemoglobin; LDL‐C, low‐density lipoprotein; LYMPH%, lymphocyte percentage; MCH, mean corpuscular hemoglobin; MCHC, mean corpuscular hemoglobin concentration; MCV, mean corpuscular volume; MONO%, monocyte percentage; NEUT#, neutrophil; NEUT%, neutrophil percentage; PCT, plateletcrit; PLT, platelet count; RBC, red blood cell Count; TBIL, total bilirubin; TC, total cholesterol; TG, triglycerides; UA, uric acid; U‐pH, urine pH value; U‐SG, urine specific gravity; WBC, white blood cell count; γ‐GT, gamma‐glutamyl transferase.

### 3.7 Two Doses of D‐Allulose Had Reduced Effects on ALP, γ‐GT, TC and HDL‐C

**FIGURE 9:**
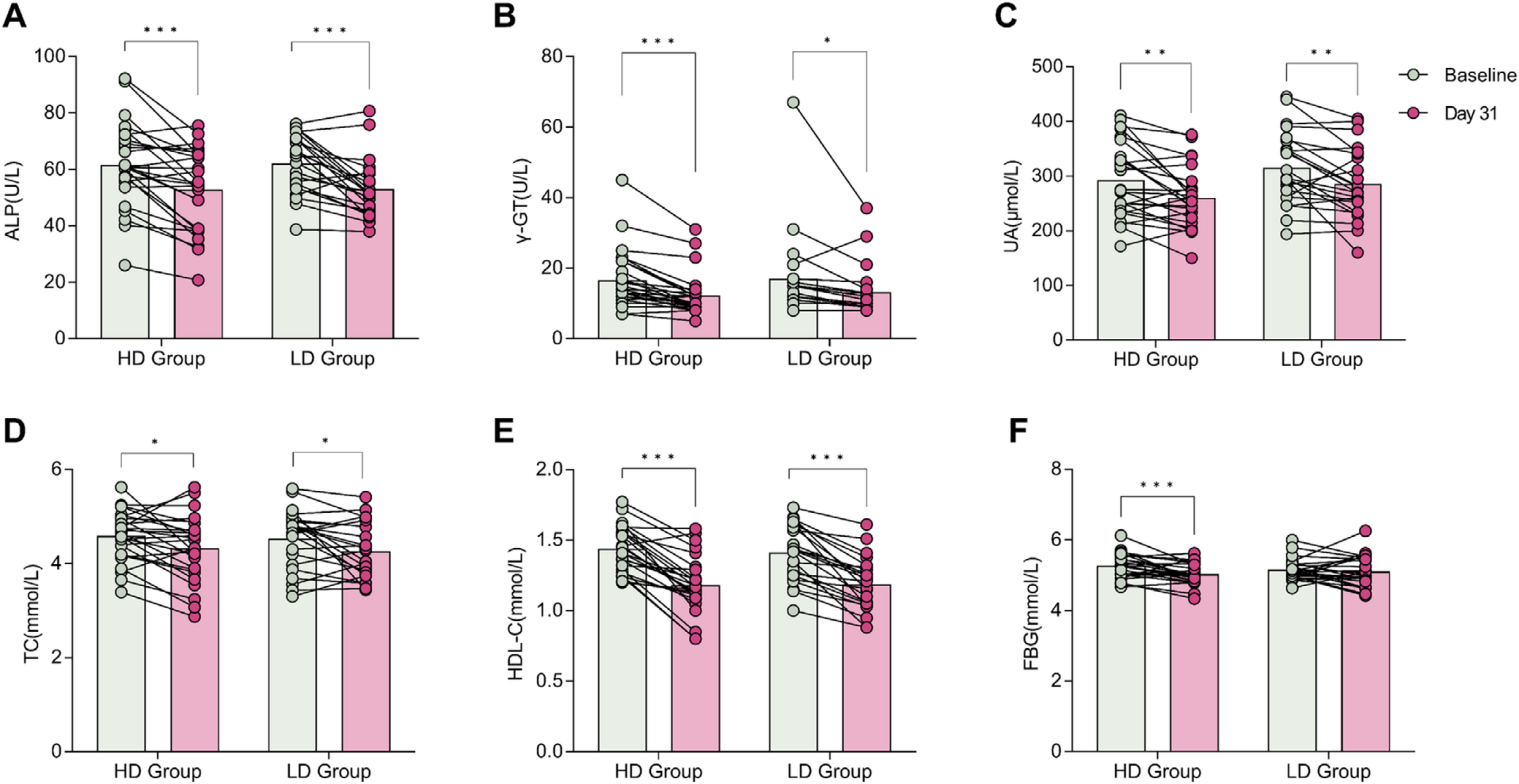
Changes in serum biochemical indices in high‐dose (HD) and low‐dose (LD) *D‐*allulose groups at baseline and after a 31‐day intervention. (A–H) show levels of serum alkaline phosphatase (ALP, A), gamma‐glutamyl transferase (γ‐GT, B), uric acid (UA, C), total cholesterol (TC, D), high‐density lipoprotein cholesterol (HDL‐C, E), and fasting blood glucose (FBG, F). Measurements are presented for both HD and LD groups at baseline and post‐intervention. Gray dots represent baseline samples; pink dots indicate samples from Day 31. Each dot represents an individual participant (HD group, *n* = 26; LD group, *n* = 23). Data are expressed as mean ± standard error of the mean (SEM). Statistical significance compared to baseline is indicated as follows: *p < 0.05, **p < 0.01, ***p < 0.001 (paired *t* test).

As stated above, no CS were detected among participants. After 30‐day *D*‐allulose intervention. Several serum biochemical indices showed statistically significant changes in both groups when compared to baseline following *D*‐allulose intake (Table 4). Specifically, significant decreases were observed in alkaline phosphatase (ALP) (*p* < 0.001 for both groups, Figure 9A), gamma‐glutamyl transferase (γ‐GT) (HD group, *p* < 0.001; LD group, *p* = 0.015, Figure 9B), uric acid (UA) (HD group, *p* = 0.003; LD group, *p* = 0.007, Figure 9C), total cholesterol (TC) (HD group, *p* = 0.015; LD group, *p* = 0.030, Figure 9D), and high‐density lipoprotein cholesterol (HDL‐C) (*p* < 0.001 for both groups, Figure 9E). All post‐intervention values remained within the normal clinical reference ranges.

Furthermore, from baseline to Day 31, only the high‐dose group had a significant reduction in fasting blood glucose (FBG) (*p* = 0.001, Figure 9F), with no significant intergroup difference between HD and LD groups (*p* = 0.180, Table 5), whereas the low‐dose group also exhibited a significant reduction in total bilirubin (TBIL, *p* = 0.035) also decreased significantly (Table 4). Other parameters, such as liver function markers alanine aminotransferase (ALT), aspartate aminotransferase (AST) and renal function parameters like creatinine, showed no significant alterations. Overall, all variations in these indices remained within the normal reference ranges, and no clinically abnormal changes were observed (Table 4).

The difference in serum biochemical indicators before and after intervention between the two dose groups were compared. The results showed that there were no statistically significant differences in the changes of all indicators between the groups (*p* > 0.05) (Table 5). Both groups showed a relatively consistent trend of changes: a decrease in alkaline phosphatase (ALP) (HD group, −8.85 ± 9.475 U/L; LD group, −9.20 ± 9.497 U/L, *p* = 0.938), a decline in gamma‐glutamyl transferase (γ‐GT) (HD group, −4.4 ± 3.99 U/L; LD group, −3.7 ± 6.64 U/L; *p* = 0.358), a reduction in uric acid (UA) levels (HD group, −31.9 ± 47.00 μmol/L; LD group, −30.1 ± 46.51 μmol/L; *p* = 0.408), a decrease in total cholesterol (TC) (HD group, −0.262 ± 0.5021 mmol/L, LD group, −0.265 ± 0.5406 mmol/L; *p* = 0.898), and a decrease in high‐density lipoprotein (HDL‐C) (HD group, −0.256 ± 0.1676 mmol/L; LD group, −0.224 ± 0.1496 mmol/L; *p* = 0.591). Fasting blood glucose (FBG) decreased (HD group, −0.239 ± 0.3119 mmol/L; LD group, −0.057 ± 0.4248 mmol/L; *p* = 0.180) (Table 5). The comparison of changes in indicators from baseline to Day 31 between the two groups showed that both doses of *D*‐allulose had similar effects on biochemical indicators.

### 3.8 High‐Dose D‐Allulose Reduces BMD T‐Scores in Participants Aged ≥ 35 Years

**FIGURE 10:**
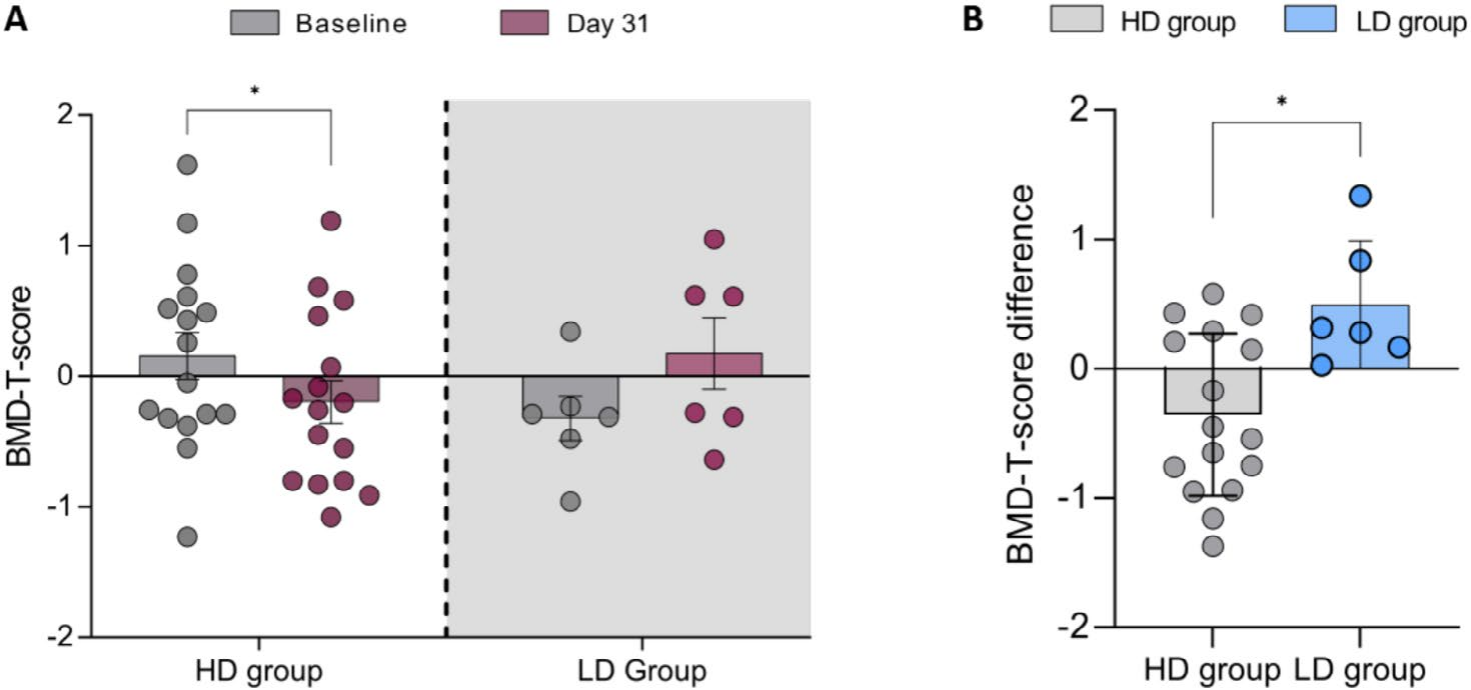
The BMD T‐score change. (A) The BMD T‐score change of participants from baseline to Day 31 in two groups. Each data point represents an individual participant (HD group, *n* = 16; LD group, *n* = 6). Change from baseline is presented as mean ± standard error of the mean (SEM). *p < 0.05: Significant difference shown at the x‐axis compared with baseline (paired t‐test). (B) The BMD T‐score difference change of participants from baseline to Day 31 between two groups (HD group, *n* = 16; LD group, *n* = 6). *p < 0.05: Significant difference shown at x‐axis between two groups (ANCOVA with baseline as a covariate).

Compared to the baseline, the BMD T‐score of subjects aged ≥ 35 years old in the high‐dose group significantly decreased after consuming the test sample. In the high‐dose group, the mean decrease in BMD T‐score was −0.354 (Table 3), which exceeded the instrument's least significant change (LSC = 0.1), confirming a true measurement change beyond random error. In contrast, the low‐dose group showed a non—significant decrease (Tables 2 and 3, Figure 10A). In addition, there was a statistically significant difference in the change of the BMD T‐score (for subjects over 35 years old) from baseline to Day 31 between the two groups (*p* < 0.05, Figure 10B and Tables 2 and 3). The high‐dose group showed a downward trend in BMD T‐score (−0.354 ± 0.6293), while the low‐dose group showed an upward trend (0.497 ± 0.4963).

### 3.9 Adverse Events

There were no significant adverse events (SAEs) observed in this study. A total of 11 adverse events (AEs), representing 22.0%, were reported. These symptoms included diarrhea, cold, headache, nausea, fullness, prolonged QT interval, elevated creatine kinase, bloating, and oral odor. Among them, 5 cases (19.2%) were documented in the high‐dose group, and 6 cases (25.0%) in the low‐dose group. The incidence of adverse events did not differ significantly between the two dose groups (*p* > 0.05), as shown in Table S3. The study investigators judged 4 of the 11 AEs to be unrelated, which included 2 cases of cold, 1 case of prolonged QT interval, and 1 case of elevated creatine kinase. The remaining 7 cases were possibly related. Apart from 1 case of cold (in the low‐dose group), which was a moderate adverse event, the others were mild. No targeted interventions were required for these adverse events. The most of mild AEs resolved spontaneously within 1–3 days without targeted intervention; however, one case of prolonged QT interval—deemed unrelated to the test product's mechanism—failed to recover even 12 days after discontinuation of *D*‐allulose intake. None of the adverse events resulted in participant withdrawal from the trial, nor were there any serious adverse events. Laboratory tests conducted on subjects who experienced adverse events after the test sample period showed all indicators within normal range.

## 4 Discussion

This trial enrolled a total of 50 subjects, with 26 assigned to the high‐dose group and 24 to the low‐dose group. One subject in the low‐dose group withdrew from the trial due to loss of follow‐up. Consequently, this high completion rate (98.0%, 49/50) attests to excellent participant compliance and the feasibility of the trial protocol.

The findings of the questionnaire survey (1388 responses in total) indicated that the incidence of GI symptoms was relatively low. These reactions were primarily characterized by bloating (5.2%), a sense of fullness (3.5%), decreased appetite (2.2%), diarrhea (1.9%), abdominal colic (1.8%), headache (1.5%), and nausea (1.0%). Most of these GI symptoms were mild. Only one case of abnormal defecation frequency was reported, and the vast majority of defecation‐related symptoms were reported on the 1st to 3rd days after the first intake. There were no statistically significant differences between the two groups in terms of indicators such as GI symptoms and abnormal defecation (*p* > 0.05). This suggests that at these doses, *D*‐allulose demonstrated good gastrointestinal tolerance, which was generally consistent with the adverse reactions and incidence rates reported in previous literature within the dose range of 0.2–0.3 g/kg body weight of *D*‐allulose (Han et al. 2018). The mechanism by which *D*‐allulose causes GI symptoms may be attributed to the following aspects. As a novel low‐calorie sweetener, approximately 70% of *D*‐allulose is absorbed by the small intestine and then excreted in the urine, without being involved in energy metabolism. The remaining approximately 30% enters the colon (Iida et al. 2010; Whistler et al. 1974). The accumulation of such low‐digestible carbohydrates in the intestine may increase the intraluminal osmotic pressure, leading to the influx of water and causing diarrhea. On the other hand, although *D*‐allulose belongs to low fermentable sugars (Matsuo 2003), some unabsorbed components are still fermented by microorganisms in the colon to produce gas. This may be the main mechanism underlying bloating and a feeling of fullness. Similar effects are also commonly observed in other low‐digestible carbohydrates (Adrienne and Chey 2017; Grabitske and Slavin 2009), and their occurrence is closely related to the intake dose.

The laboratory test results in this study demonstrated that all parameters—hematological examination, serum biochemical testing, urinalysis and fecal analysis—remained within the normal physiological range following the feeding trial. No clinically significant (CS) abnormalities were detected. Moreover, no obvious abnormal changes were observed in the main vital signs as well as the vast majority of physical parameters. These findings confirm that within the tested dosage range, *D*‐allulose did not cause systemic toxic or side effects, and its overall safety was controllable.

However, following a 30‐day population intervention study, several blood and serum biochemical indicators exhibited consistent statistical changes post‐intervention, particularly concerning the parameters of the red blood cell and platelet systems. The RBC and HCT in both dose groups showed a decrease, while MCH and MCHC demonstrated an increase. Concurrently, there was also a significant reduction in PLT across both groups. In terms of serum biochemistry, ALP, γ‐GT, uric acid, TC, and HDL‐C all experienced declines; notably, fasting blood glucose levels in the high‐dose group also significantly decreased. Among these alterations, changes in ALP, HDL‐C, and FBG were consistent with findings from previous human studies (Tanaka et al. 2020, 2019). The observed reduction in HDL‐C, while statistically significant, remained within the normal clinical reference ranges. The clinical significance of this change is unclear, as the 30‐day intervention period is too short to infer long‐term cardiovascular effects, and no adverse events related to lipid metabolism were observed. Therefore, this change should be interpreted as a physiological adaptation rather than an adverse signal. Future longer‐term studies are needed to clarify the implications of HDL‐C modulation by *D*‐allulose. Regarding fasting blood glucose, although FBG decreased significantly only in the high‐dose group, the lack of a significant between‐group difference indicates that this finding should not be interpreted as a clear dose‐dependent effect.

Although all observed changes remained within clinical normal ranges, their consistency and statistical significance suggest that *D*‐allulose may exert regulatory effects on three key physiological processes: the hematopoietic system, glycolipid metabolism, and liver enzyme activity. Analyzing the underlying mechanisms suggests that *D*‐allulose may modulate the body's metabolic environment indirectly influencing hematopoiesis. Research indicates that metabolic state serves as a crucial regulatory factor for differentiating hematopoietic stem cells and progenitor cells (Ito and Suda 2014; Oburoglu et al. 2014). The alterations in glycolipid metabolism induced by *D*‐allulose—such as reductions in FBG and HDL‐C—may systematically fine‐tune erythroid and megakaryocytic lineage generation efficiency through energy‐sensing pathways like AMPK (Garcia and Shaw 2017; Hattangadi et al. 2011). However, it is important to note that this phenomenon might represent a physiological adaptation rather than a toxicological response.

It is important to note that some of the aforementioned results are inconsistent with data from animal experiments. This study observed a decrease in PLT and ALP levels, while a 90‐day toxicity test in rats reported significant increases in both indicators (An et al. 2019; Kanako and Tatsuhiro 2009). This interspecies discrepancy likely relates to species‐specific absorption and metabolism of *D*‐allulose, which is crucial for the experimental system. Rats and humans show differences in *D*‐allulose absorption efficiency: rats absorb approximately 20%–30% of orally administered *D*‐allulose (Matsuo 2003), whereas humans exhibit an absorption rate of 66%–79% (Iida et al. 2010). This difference stems from species‐specific intestinal transporters (e.g., glucose transporter (GLUT) isoforms) and metabolic clearance patterns—rats metabolize part of the absorbed *D*‐allulose (Kishida et al. 2023), while humans excrete most unmetabolized *D*‐allulose via urine (Iida et al. 2010). The unabsorbed fraction further influences short‐chain fatty acid (SCFA) production: extensive cecal fermentation in rats generates abundant SCFAs, activating intestinal epithelial cells to elevate ALP and stimulate thrombopoietin secretion to increase PLT (Reese et al. 1985; Higueras et al. 2025). In contrast, minimal SCFA production in humans due to high *D*‐allulose absorption does not trigger similar effects on ALP or PLT (Basson and Sgambati 1998); instead, the absence of SCFA‐mediated regulatory signals may lead to reductions in these two indicators.

Furthermore, high‐dose *D*‐allulose significantly reduced bone mineral density (BMD) T‐scores in participants over 35, showing a notable inter‐group difference compared to the low‐dose group (*p* < 0.05). This is probably linked to two mechanisms related to human ALP. First, humans absorb about 66%–79% of *D*‐allulose (Iida et al. 2010), leaving little substrate for colonic fermentation and SCFA production. SCFAs like butyrate are crucial for the gut‐bone axis; their deficiency disrupts G protein‐coupled receptor 41 (GPR41)/insulin‐like growth factor 1 (IGF1) signaling pathway (Xiao et al. 2022), impairing osteoblast proliferation and differentiation. Second, low SCFA levels fail to inhibit histone deacetylases, reducing Runt‐related transcription factor 2 (Runx2)/Osterix (SP7) expression and suppressing osteoblastic ALP synthesis. Since ALP is vital for bone mineralization (Sun et al. 2025; Li et al. 2024), its dysfunction hinders bone formation. It is important to note that a 30‐day period is insufficient to capture genuine bone remodeling, which typically takes months to years. Hence, the observed reduction in BMD T‐score more likely represents a transient physiological adaptation rather than sustained bone loss. In contrast, the low‐dose group showed a non‐significant increase in BMD, likely due to mild SCFA‐induced protective effects on bone health. Notably, the decrease in BMD T‐score in the high‐dose group remained within the normal range, with no participants developing osteopenia (T‐score < −1.0), indicating that the change is unlikely to pose immediate clinical risks. Given the 30‐day intervention duration, modest sample size, and restriction to healthy normal‐BMI adults, the safe intake recommendation should be interpreted cautiously. Based on these findings, a provisional safe intake limit of 0.4 g/kg bw/day (≈24 g/day for a 60 kg adult) is proposed for this population, and longer‐term and larger‐scale studies are required to validate this threshold and to assess its applicability to other populations.

## 5 Conclusions

This 30‐day randomized, double‐blind, parallel‐group design with pre‐post comparison trial evaluated the safety, tolerance, and physiological effects of *D*‐allulose in healthy Chinese adults. The results confirm that *D*‐allulose has good gastrointestinal tolerance: GI symptoms were mild, transient (primarily during Days 1–3 of intervention), and showed no dose‐dependent differences, aligning with the safety profile of low‐digestible carbohydrates. All systemic safety indicators (hematology, serum biochemistry, urinalysis, vital signs, and body composition) remained normal, indicating no systemic toxic side effects at tested doses. Beyond basic safety, *D*‐allulose demonstrated consistent physiological regulatory effects: it modulated hematopoietic parameters (decreased RBC, HCT, PLT; increased MCH and MCHC) and altered glycolipid metabolism indicators (decreased ALP, γ‐GT, UA, TC, HDL‐C, and FBG in the high‐dose group). These alterations imply that *D*‐allulose may exert systemic regulatory effects via energy metabolism pathways such as AMP‐activated protein kinase (AMPK), and such changes are more likely to reflect physiological adaptation rather than adverse toxic responses. Notably, this study found that high‐dose *D*‐allulose (36 g/day) significantly reduced BMD T‐scores in participants aged ≥ 35 years compared to the low‐dose group. This previously unreported effect may be linked to insufficient short‐chain fatty acid production due to high *D*‐allulose absorption in humans affecting gut‐bone axis and osteoblast function. Additionally, species‐specific differences were noted: unlike rat studies showing increased PLT and ALP levels, human data revealed decreases attributed to divergent absorption efficiency and SCFA‐mediated regulatory discrepancies. Considering the relatively short intervention duration, modest sample size, and limited study population (healthy normal‐BMI Chinese adults), a provisional safe intake limit of 0.4 g/kg bw/day (≈24 g/day for a 60 kg adult) is recommended only for this population; further long‐term studies in broader age groups and diverse populations are required to validate this provisional threshold and to further evaluate potential bone health effects.

## Author Contributions

**Nan Zhang:** methodology, writing – review and editing. **Dan Li:** investigation. **Haiming Jing:** supervision, resources. **Shan Gao:** conceptualization, methodology, data curation, funding acquisition, project administration, resources, writing – review and editing. **Jiangli Han:** methodology, investigation, resources. **Fangfang Wang:** investigation. **Zinan Li:** validation, supervision, writing – review and editing. **Yanmin Nie:** supervision, writing – review and editing. **Junyu Ning:** methodology, funding acquisition, resources, project administration. **Lijuan Qi:** conceptualization, methodology, software, validation, investigation, data curation, formal analysis, visualization, writing – original draft. **Wenjing Zhang:** methodology, software, investigation, validation, writing – review and editing.

## Funding

This work was supported by the Beijing Municipal Science and Technology Commission, Administrative Commission of Zhongguancun Science Park (Grant No. Z231100004523001).

## Ethics Statement

This study was conducted with approval from the Institutional Review Board or Ethics Committee at Beijing Center for Disease Prevention and Control, Beijing, China and Peking University Third Hospital. This trial was registered as ChiCTR2500100349 (http://www.chictr.org.cn).

## Consent

Informed consent was obtained from all subjects involved in the study.

## Conflicts of Interest

The authors declare no conflicts of interest.

## Supporting information


**Table S1:** Quality control data of three consecutive *D*‐allulose batches.
**Table S2:** Specific medical examination indicator profiles of participants (Related to Table 1).
**Table S3:** Adverse events reported by participants in the *D*‐allulose intervention trial.

## Data Availability

The data that support the findings of this study are available from the corresponding author upon reasonable request.
