# Supplementary material for: D‐Allulose as a Low‐Calorie Sweetener: A 30‐Day Randomized, Double‐Blind Study on Gastrointestinal Tolerance and Systemic Safety to Support Its Application in Healthy Diets
Source: Food Sci Nutr. 2026 Jun 29;14(7):e72042. doi: 10.1002/fsn3.72042 (PMC13312298; doi:10.1002/fsn3.72042)
Supplement: Supplementary file 1 — Table S1: Quality control data of three consecutive D‐allulose batches. Table S2: Specific medical examination indicator profiles of participants (Related to Table 1). Table S3: Adverse events reported by participants in the D‐allulose intervention trial. [file FSN3-14-e72042-s001.doc]

**Supplemental Table 1**

Quality control data of three consecutive *D*-allulose batches.

| **Batch Number** | **Purity (%)** | **Endotoxin (EU/mg)** | **Residual Proteins (mg/kg)** |
| --- | --- | --- | --- |
| CAIQS0024003721001 | >99.0 | ＜10 | N.D. (Not Detected) |
| CAIQS0024003721002 | >99.0 | ＜10 | N.D. |
| CAIQS0024003721003 | >99.0 | ＜10 | N.D. |
| *The purity of *D*-allulose was determined via HPLC according to the specifications established by the National Health Commission of China (NHC Announcement, July 2025). Endotoxin levels (gel clot method) and residual protein content (Coomassie Brilliant Blue staining) were assessed following the protocols described in the NHC regulatory standards (October 2023). | | | |

**Supplemental Table 2**

Specific medical examination indicator profiles of participants (Related to Table 1).

|  | **HD group (n=26) (%)** | **LD group (n=24) (%)** | **Total (n=50) (%)** |
| --- | --- | --- | --- |
| T-βHCG |  |  |  |
| 0 | 11(42.3) | 11(45.8) | 22(44.0) |
| 1 | 0 | 0 | 0 |
| 2 | 0 | 0 | 0 |
| Upper abdomen ultrasonography |  |  |  |
| 0 | 16(61.5) | 16(66.7) | 32(64.0) |
| 1 | 10(38.5) | 8(33.3) | 18(36.0) |
| 2 | 0 | 0 | 0 |
| Chest X-ray |  |  |  |
| 0 | 24(92.3) | 20(83.3) | 44(88.0) |
| 1 | 2(7.7) | 4(16.7) | 6(12.0) |
| 2 | 0 | 0 | 0 |
| T-βHCG, total beta-human chorionic gonadotropin (for female only), 0=normal; 1=abnormal but without clinical significance;2=abnormal with clinical significance. | | | |

**Supplemental Table 3**

Adverse events reported by participants in the *D*-allulose intervention trial.

|  | **HD group (N=26)** | **LD group (N=24)** | **P value** |
| --- | --- | --- | --- |
| **AE (number, n%)** | 5(19.2) | 6(25.0) | 0.487 |
| **SAE (number, n%)** | 0(0.0) | 0(0.0) | - |
| **Adverse Event Category (number, n%)** | | | |
| Diarrhea | 2(7.7) | 1(4.2) | - |
| Common cold | 1(3.8) | 1(4.2) | - |
| Headache | 0(0.0) | 1(4.2) | - |
| Nausea | 0(0.0) | 2(8.3) | - |
| Satiety | 0(0.0) | 2(8.3) | - |
| Abdominal distension | 0(0.0) | 1(4.2) | - |
| QT prolongation | 1(3.8) | 0(0.0) | - |
| Creatine kinase increased | 1(3.8) | 0(0.0) | - |
| Halitosis | 0(0.0) | 1(4.2) | - |
| AE, adverse events;SAE, serious adverse events. | | | |
